# Josephson radiation threshold detector

**DOI:** 10.1038/s41598-024-52684-2

**Published:** 2024-01-30

**Authors:** Soragga Ali, P. H. Ouyang, J. X. He, Y. Q. Chai, L. F. Wei

**Affiliations:** Information Quantum Technology Laboratory, International Cooperation Research Center of China Communication and Sensor Networks for Modern Transportation, School of Information Science and Technology, Southwest Jiaotong University, Chengdu, 610031 China

**Keywords:** Single photons and quantum effects, Experimental particle physics

## Abstract

A Josephson radiation threshold detector (JRTD) that is based on the threshold behaviour of a current bias Josephson junction (CBJJ) is designed and fabricated for infrared radiation (IR@1550nm) detection at low temperatures. To achieve the optimal performance, we develop a binary hypothesis detection method to calibrate Josephson threshold behaviours (i.e. the switching current distributions of the CBJJ with the Al/AlO_x_/Al junction) in the absence and presence of radiation. In the absence of IR radiation, the junction transitioned with a measurable voltage drop across the junction, and this signal was treated as the events of hypothesis H_0_. The events of junction transition observed in the presence of the IR radiation served as hypothesis H_1_. Considering the usual Gaussian noise and based on statistical decision theory, the accumulated data of the measured switching current distributions are processed, and the threshold sensitivity of the demonstrated JRTD device is estimated. The minimum detectable IR radiation power of the proposed detector is approximately 0.74 pW, which corresponds to the photon rate of 5.692 × 10^6^ photons/second. Further optimisation of JRTDs to implement the desired binary detection of a single photon is still a subject of argument, at least theoretically.

## Introduction

The development of superconducting electronic devices has facilitated the very sensitive detection of photons and particles. Desirably, the single-photon detectors for different practical applications are expected to possess (i) a wide waveband response, (ii) a shorter response time, (iii) higher quantum efficiency^1–4^ and the ability to count single photons^5^. Amongst various devices that demonstrate single-photon detection, the use of superconducting Josephson junctions (JJs) as single-photon detectors is particularly desirable and have been investigated extensively owing to their theoretically good energy resolution, high responsivity and high count-rate capabilities^6–10^. Physically, if the energy of the applied photon is larger than the superconducting gap, then Cooper pairs in the superconductor can be broken, and the quasiparticles can be generated consequently. This phenomenon might lead to relevant observable effects and thus can be utilised to implement the detection of illuminating photons. Indeed, the JJ-based axion detection^11–13^, superconducting nanowire single-photon detectors^14–19^, transition-edge sensors^20–22^, microwave kinetic inductance detectors^23,24^ and the superconducting qubit detectors^25–28^, are under development for astronomic observations, axion searches^29–35^, quantum optical communication^36,37^ and optical quantum computation^38,39^.

Physically, any radiation-induced threshold behaviour of the device can be utilised to generate the photon detectors^40–44^ (i.e. the threshold detector (TD)). These devices can possess numerous notable features^45,46^, including their ability to operate at high speeds, minimal resource demands and high efficiency in detecting signals. In fact, this technology has been applied in multisensor networks or distributed intelligent systems^41,43^, as well as weak sinusoidal signal detection in marine environment^44^. For example, a maximum posteriori probability detector demonstrated in Ref.^41^ utilised two-state threshold nonlinearity. Even in the presence of non-Gaussian noise, the TD can enhance the performance of signal detection compared with the linear detector^47^. Basically, threshold detection benefits from the ability to determine the existence of a signal by observing the shifts from one metastable condition to another^48,49^. Specifically, the JRTD possesses (i) a binary capability to distinguish between two states with or without the radiation; (ii) a feasible technique to trigger the transition from one state to the other and (iii) the possibility to identify these transitions from the superconducting-to-resistive state by observing whether the junction switching current behaviours cross the detector threshold level or not^50^. A CBJJ can be depicted as a phase particle trapped in a metastable state of a cosine potential^28,51,52^; it can ‘escape’ if the bias current, I_b_, approaches the critical current of the JJ. As a consequence, a switching event (i.e. from the zero-voltage state to a finite-voltage state) can be observed, and thus, relevant physical quantities, such as the switching currents, can be measured^53,54^. Due to the presence of Gaussian noise, the switching current measurement should show certain statistical distributions. CBJJs provide a feasible platform to investigate macroscopic quantum tunnelling (MQT)^55,56^, thermal activation (TA)^57^ and phase diffusion^58,59^. Therefore, by monitoring the changes in these statistical distributions under the applied IR radiation power, CBJJ can be regarded as a threshold detector to implement photon detection^4,60^. JJs can be regarded as JRTD detectors that provide a readily observable signal when exposed to a disturbance of radiation energy that is equivalent to a typical threshold^61,62^. Indeed, the CBJJ-based photon detectors, including the Josephson escape detectors^63,64^, graphene-based CBJJ detectors^9,65,66^ and microwave single-photon detectors^67–69^, have received extensive attention in recent years. Indeed, the JJ serves as an intrinsic threshold detector that has been applied to implement the current fluctuation detection^70–72^. The test methodology described in^73^ utilises the declared temperature dependence of the critical currents of JJs, which must still be optimised for the sensitive radiation detection.

In this paper, based on binary detection theory, we designed and fabricated the JRTD to demonstrate IR@1550nm radiation detection at low temperatures. We address the issues of the Josephson radiation threshold circuit (JRTC) to solve the fast binary hypothesis detection problem. We optimise the proposed JRTD through the analysis of the experimentally measured switching current distributions of the (Al/AlO_x_/Al) CBJJ. In the absence of IR radiation (i.e. dark count), the bias current I_b_ is gradually increased from 0–25 µA, the junction transition from superconducting-to-resistive state with a measurable voltage drop across the junction can be observed, and the switching current distribution mimics hypothesis H_0_. Meanwhile, in the presence of an IR@1550nm radiation with the power being 740 nW, the junction transition from superconducting-to-resistive state is observed again, and the switching current distributions mimics hypothesis H_1_. Considering the effects of Gaussian noise, a method based on standard statistical decision theory is developed to process the accumulated data of the switching current distributions to estimate the threshold control functions, namely, Th_1_ and Th_2_, and thus the detection sensitivity. Furthermore, the numerical processing of the detected data can be further optimised to improve the quality of the detector.

## Model and method

**Figure 1:**
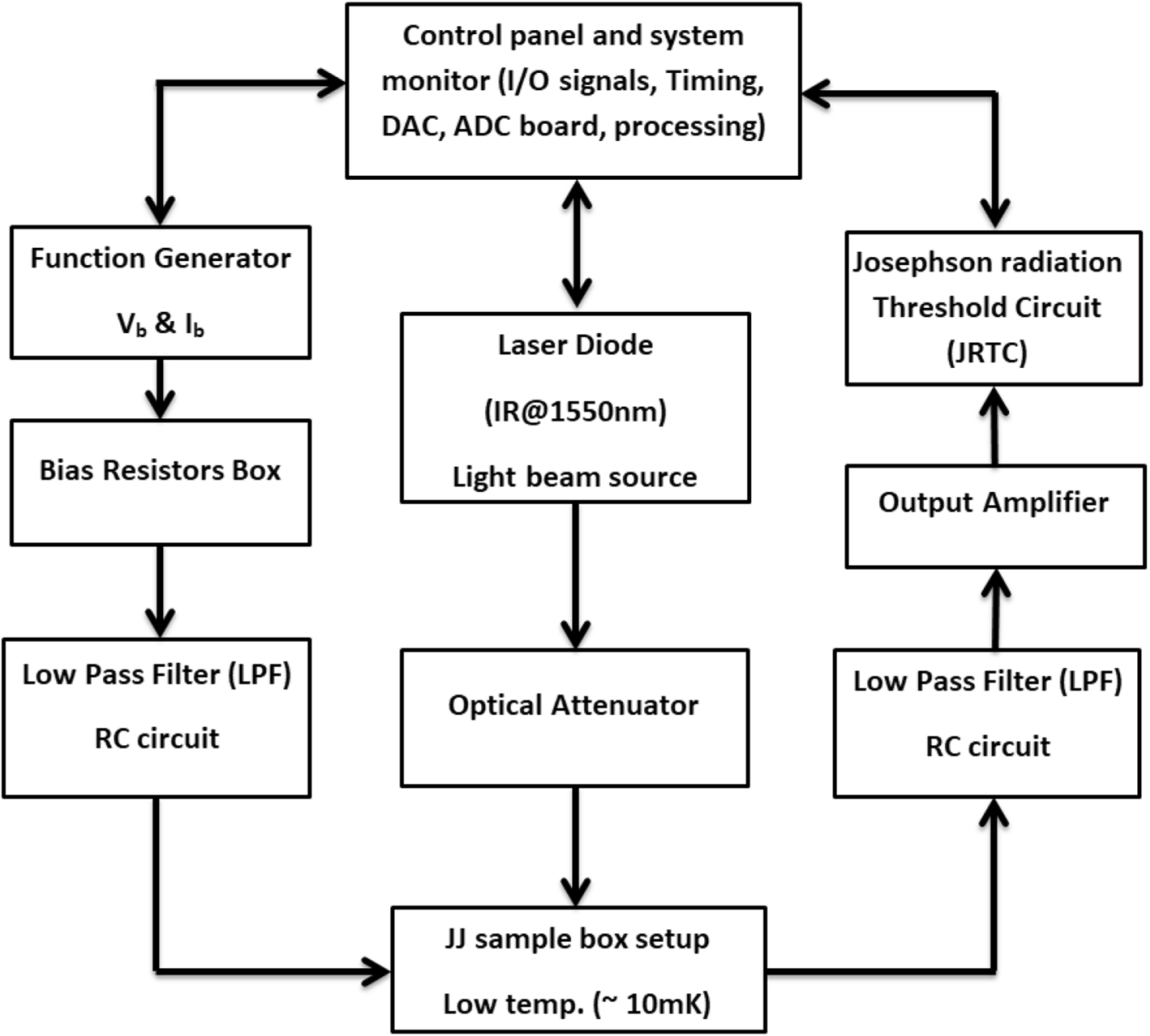
Schematic of the JRTD detector.

The CBJJs are used to implement the binary detection^74^ of the infrared photons. Figure 1 illustrates the components of the JRTD and provides a comprehensive explanation of the control mechanisms for governing the input/output signals (e.g. the bias voltage, current, resistance and waveforms), as well as the characteristics of the IR@1550nm radiation power and the threshold switching current value. The JRTD operation is presented as follows: Initially, let the CBJJ be biased at I_b_ < I_sw_. Next, increase the bias current gradually until the junction transition with a measurable voltage drop can be observed across the junction^75^. This state mimics hypothesis H_0_. Then, illuminate the device with the IR@1550nm radiation power via the fibre focus and increase the bias current gradually until the junction transition with a measurable voltage drop can be observed across the junction again. This state mimics hypothesis H_1_. The input and output signals are passed via a low-pass filter (LPF) to separate the noise component. Subsequently, the output signal is amplified by a power amplifier with a gain of 10^3^ before being inputted into the JRTC for the binary hypothesis detection process. The measurement of the switching current distributions of the CBJJ is conducted by carefully probing the abrupt transition from the zero-voltage state to a non-zero finite voltage state. This transition is also influenced by the fluctuations in TA and MQT. Certainly, under radiation power, the absorbed IR@1550nm illumination would break the Cooper pairs in the superconductor and thus generate quasiparticles, which might reduce the switching current compared with the first case.

### CBJJ’s detection

**Figure 2:**
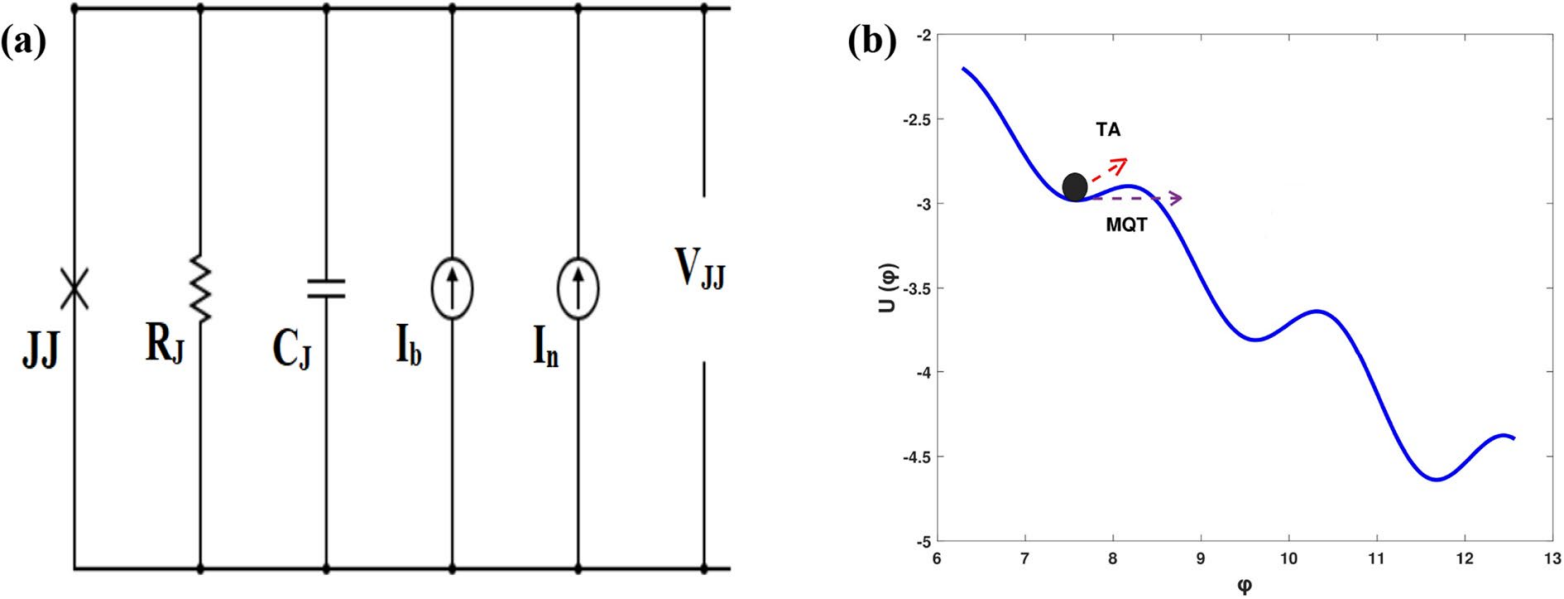
CBJJ is described by: (**a**) RCSJ circuit model with the bias current, I_b_, and noise current, I_n_; (**b**) Physical model of a phase particle moving in a minimum of the potential U (φ) in the noisy environment.

Firstly, let the CBJJ be a biased current I_b0_, which is far from the switching current, and the voltage across the junction is kept at zero state. As seen in Fig. 2a, a current-biased JJ can be described by a resistively and capacitively shunted junction (RCSJ) circuit model^76^, which consists of four parallel components, namely, resistor, capacitor, pure JJ and the current sources I_b_ and I_n_ being the bias and noise currents, respectively. Also, as shown in Fig. 2b, the CBJJ can be described physically by a phase particle trapped in a minimum of the potential^28,77^ $;{\mathrm{ U}\left(\varphi \right)=-{\text{E}}}_{{\text{J}}}[{\text{cos}}\varphi +({{\text{I}}}_{{\text{b}}}/{{\text{I}}}_{{\text{c}}})\mathrm{\varphi }]$, where U(φ) is the washboard potential, E_J_ is the JJ coupling energy, φ is the phase difference across the junction, and I_c_ is the critical current of the JJ.

The dynamics of such phase particle can be described by the following 2nd-order differential equation:

1 $${{\text{C}}}_{{\text{J}}}\frac{\mathrm{\hbar }}{2{\text{e}}}\frac{{{\text{d}}}^{2}\varphi }{{{\text{dt}}}^{2}}+\frac{1}{{{\text{R}}}_{{\text{J}}}}\frac{\mathrm{\hbar }}{2{\text{e}}}\frac{{\text{d}}\varphi }{{\text{dt}}}+{{\text{I}}}_{{\text{c}}}{\text{sin}}\varphi ={{\text{I}}}_{{\text{b}}}+{{\text{I}}}_{{\text{n}}}.$$

**Figure 3:**
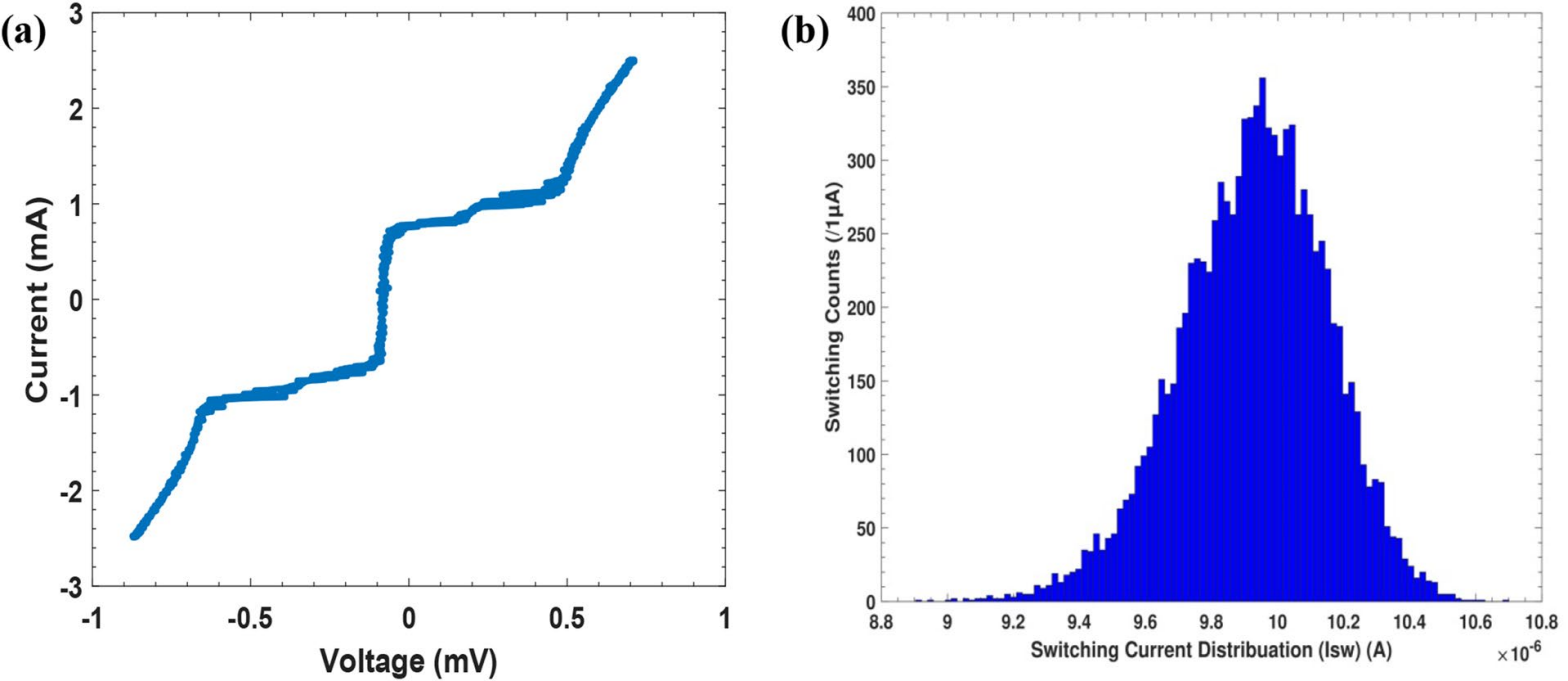
Experimental measurements of the fabricated CBJJ by gradually changing the bias current I_b_: (**a**) the I-V curve and (**b**) the distribution of the switching current I_sw_.

This equation is a Langevin-type calculation with stochastic noise current, I_n_^71,78^, where ħ = h/2π is the reduced Planck constant; e is the electron charge; and C_J_ and R_J_ are the junction capacitance and junction shunt resistance, respectively. When the bias current gradually increases from a fixed value I_b0_ to a certain value I_sw_ within the duration t, the phase particle can escape, either through TA or MQT^79^, from the minimum potential barrier. Thus, a finite voltage state can be observed across the junction. The (I–V) curve of the JJ is measured by connecting the JJ with a variable AC power supply (function generator). Suppose that the bias current flowing through JJ is evenly distributed in the junction. When the current density is less than the critical current density j_c_, the AC Josephson effect relation ${{\text{I}}={\text{I}}}_{{\text{c}}}{\text{sin}}\varphi$ is maintained. Thus, the Cooper pairs that flow through the JJ generate the supercurrent (I) without voltage across the junction. However, if the current density is greater than the critical current density (i.e. j > j_c_), then the relation ${{\text{I}}={\text{I}}}_{{\text{c}}}{\text{sin}}\varphi$ does not hold, which means that the tunnelling particles are not the Cooper pairs but the quasiparticles, and thus a voltage drop signal appears across the junction. Therefore, the I–V curve of the CBJJ should be characterised as follows: i) if I_b_ is sufficiently less than I_c_, then a zero-voltage signal exists and ii) for I_b_ ≥ I_c_, the voltage signal shows a finite value; until the I–V relation describes the normal-state resistance behaviour^80,81^. Schematically, Fig. 3a shows the measured non-hysteretic I-V curve of the CBJJ by measuring the junction capacitance and resistance using a digital multi-meter that shows the values of C_J_ ~ 4.4 nf and R_J_ ∼ 160 Ω, respectively. Due to the presence of noise and bias currents, the measured switching current shows the relevant distribution in Fig. 3b with an estimated value (I_sw_ ~ 9.95 µA). For the above measurements without the IR illumination (i.e. dark count), the CBJJ can be applied to implement the desired weak radiation detection, in principle, by comparing the measured signals under the weak IR radiation power to those without IR radiation^4,9^.

Secondly, the device is illuminated with IR@1550 nm radiation power using the fibre focus, and the bias current is incrementally increased until a nonzero voltage signal becomes detectable. We performed numerous measurements under identical conditions. Each iteration began with a bias current that is less than I_c_ and proceeded to record the occurrence of switching current distribution, I_sw_.

### JRTC

**Figure 4:**
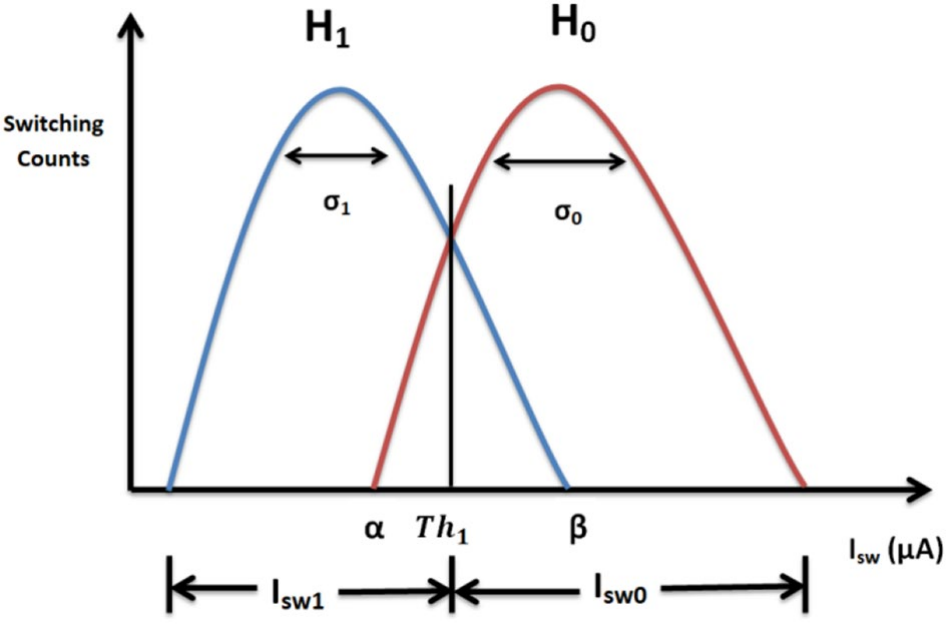
Sketch and application of the detection scheme to the average data, assumed to be Gaussian distributions of the two hypotheses: H_0_, and H_1_, with switching current distributions; I_sw0_, and I_sw1_, and standard deviations; σ_0_, and σ_1_. The threshold bound of the switching currents Th_1_ determines the quantities α, and β, the false detection probability (only the noise induces escapes), and the probability of detection IR@1550nm radiation power (the noise and the arrival of IR radiation power induce escapes), respectively.

The JRTC refers to a device that is utilised to determine the presence or absence of a finite voltage state, which is achieved by subjecting the junction current to the pre-threshold system Th_1_, followed by the aggregation of the data samples. Then, the cumulative value of these samples is assessed to ascertain whether it surpasses the threshold Th_2_ or not. The primary purpose of this circuit is to facilitate binary hypothesis detection, specifically to distinguish between H_0_ and H_1_. Figure 4 presents the binary hypothesis detection^82,83^ issue, wherein the obtained histogram exhibits compatibility with two critical outcomes, namely, the only existence of noise (referred to as hypothesis H_0_) or the occurrence of the IR@1550nm radiation power absorption within a backdrop of noise (referred to as hypothesis H_1_). The issue may be formulated using two hypotheses in a binary test^74,84^:

2 $$\left\{\begin{array}{c}{{\text{H}}}_{0} : I\left[{\text{n}}\right]= {{\text{I}}}_{{\text{sw}}}\left[{\text{n}}\right], \,\,\,\,\,\,\,\,\,\,\,\,\,\,\,\,\,\,\,\,\,\,\,n=\mathrm{0,1},\dots ,N-1\\ {{\text{H}}}_{1} : I\left[{\text{n}}\right]= {{\text{I}}}_{{\text{sw}}}\left[{\text{n}}\right]-s\left[{\text{n}}\right], \,\,\,\,\,\,\,\,n=\mathrm{0,1},\dots ,N-1.\end{array}\right.$$

The first hypothesis, denoted as H_0_ or the null hypothesis, posits that escapes are only induced by the noise. The second hypothesis, denoted as H_1_ or the alternative hypothesis, suggests that escapes are induced by noise and the occurrence of IR@1550nm radiation power. In this context, the I_sw_[n] represents the switching current value, whereas s[n] denotes the IR@1550nm radiation power. The detection scheme can be elucidated in Fig. 4, to use the Gaussian distribution function and the central limit theorem to determine the optimal decision rule for detecting the IR@1550nm radiation power.

**Figure 5:**
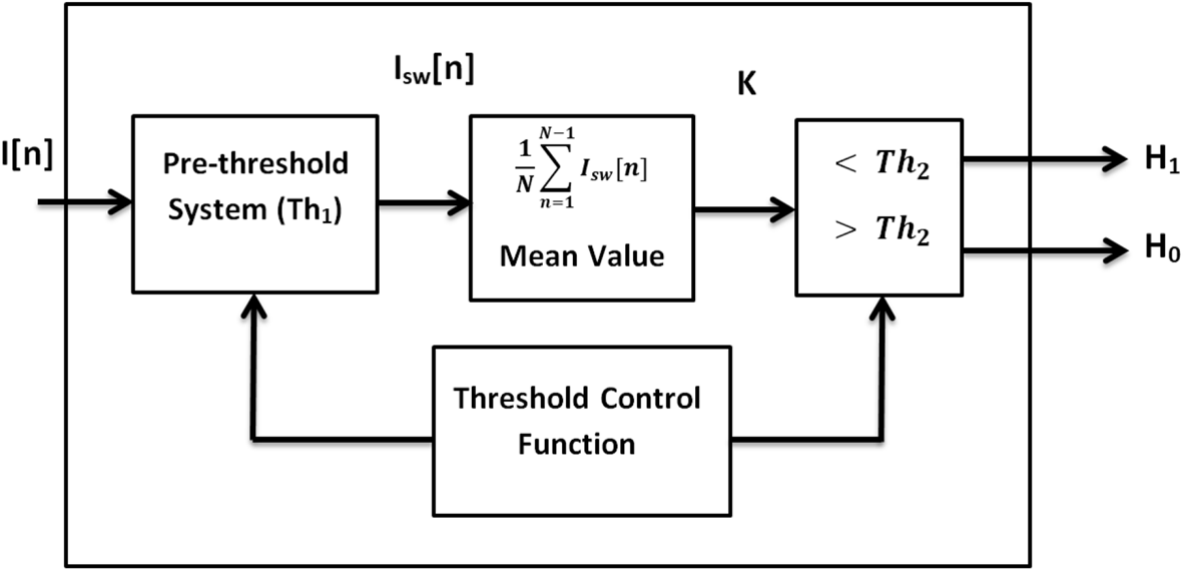
Schematic of the JRTC circuit.

Mathematically, the switching probability without radiation power P (I_sw_)_0_ and with IR radiation power P (I_sw_)_1_ can be described by:

3 $${\text{P}}{({{\text{I}}}_{{\text{sw}}})}_{0}=\frac{1}{\sqrt{2\pi }{\sigma }_{0}}exp\left(-\left(\frac{{({{I}_{sw}}_{0}-{\mu }_{0})}^{2}}{2{\sigma }_{0}^{2}}\right) \right) ,$$

and

4 $${\text{P}}{({{\text{I}}}_{{\text{sw}}})}_{1}=\frac{1}{\sqrt{2\pi }{\sigma }_{1}}exp\left(-\left(\frac{{({{I}_{sw}}_{1}-{\mu }_{1})}^{2}}{2{\sigma }_{1}^{2}}\right) \right) ,$$

respectively, where ${{I}_{sw}}_{0}$ is the switching current in the absence of IR radiation, and ${{I}_{sw}}_{1}$ is the switching current in the presence of IR radiation power. µ, σ and, σ^2^ are the mean value, standard deviation and variance of the switching current, respectively. To ascertain the switching current value, the initial step involves the calculation of the disparities between the switching probabilities with and without IR radiation power. The schematic of the JRTC used for signal detection is shown in Fig. 5. H_1_ decision is made if K < Th_2_. Note that Th_1_ and Th_2_ are different thresholds. Th_2_ is the threshold used to decide H_0_ or H_1_ (binary detection threshold), whilst Th_1_ is the pre-threshold to separate between the escape induced only by the noise and escape induced by the noise and the arrival of IR@1550nm radiation power (Fig. 4).

The JRTD performance is sensitive to Th_1_, which requires accurate computation^47,82^. Then, we can calculate the pre-threshold bound of the switching current Th_1_:

5 $${{\text{Th}}}_{1}\triangleq \left({\sigma }_{1}{{I}_{sw}}_{0}+{\sigma }_{0}{{I}_{sw}}_{1}\right)/\left({\sigma }_{0}+{\sigma }_{1}\right).$$

The false detection probability and the alternative probability limitations can be derived using the interval probability for I_sw_ being in the interval [α, β] as:

6 $${{\text{P}}}_{{{\text{I}}}_{{\text{sw}}}}\left[\alpha \le {{\text{Th}}}_{1}\le \beta \right]={\int }_{\alpha }^{\beta }\frac{1}{\sqrt{2\pi }{\sigma }_{0}}exp\left(-\left(\frac{{\left({{\text{Th}}}_{1}-{\mu }_{0}\right)}^{2}}{2{{\sigma }^{2}}_{0}}\right)\right)d{I}_{sw} = \frac{1}{2}\left[\mathit{erf}\left(\frac{\beta -{\mu }_{0}}{\sqrt{2}{\sigma }_{0}}\right)-\mathit{erf}\left(\frac{\alpha -{\mu }_{0}}{\sqrt{2}{\sigma }_{0}}\right)\right]$$

Where erf is the error function, α and β factors are related to the false detection probability of the noise that induces escapes and that of the noise and the arrival of IR@1550nm radiation power, respectively. The switching probability of the binary detection can be estimated by^85^:

7a $${{\text{H}}}_{0}: P\left[{{\text{I}}}_{{{\text{sw}}}_{0}}>\alpha \right]={\int }_{\alpha }^{\infty }\frac{1}{\sqrt{2\pi }{\sigma }_{0}}exp\left(-\left(\frac{{\left({Th}_{1}-{\mu }_{0}\right)}^{2}}{2{{\sigma }_{0}}^{2}}\right)\right)d{I}_{sw},$$

7b $${H}_{1}: P\left[{{\text{I}}}_{{{\text{sw}}}_{1}}<\beta \right]={\int }_{-\infty }^{\beta }\frac{1}{\sqrt{2\pi }{\sigma }_{1}}exp\left(-\left(\frac{{\left({\mathbf{T}\mathbf{h}}_{1}-{{\varvec{\mu}}}_{0}\right)}^{2}}{2{{{\varvec{\sigma}}}_{0}}^{2}}\right)\right)d{I}_{sw}.$$

Note that the switching current distribution (I_sw_) measurements presented here demonstrate that the detection of weak radiation is also possible when the Gaussian noise is comparable^61,86^.

In the context of the performance evaluation for a JRTD detector, the commonly used metrics include the detection probability, P_D_, and the false probability of switching, P_FS_. With regard to the provided test in Fig. 5 and the observation ${\text{I}}\left[{\text{n}}\right],\mathrm{ n}\in [0,\mathrm{ N}-1]$, calculating the test statistic K is possible. If L (K) < Th_2_, then the decision H_1_ is made, where ${\text{L}}\left({\text{K}}\right)= \frac{{f}_{K}(k;{H}_{1})}{{f}_{K}(k;{H}_{0})}$ is the likelihood ratio test on K, and ${f}_{K}\left(k;{H}_{i}\right), i=\mathrm{0,1}$ represents the probability density functions of K according to the two hypotheses (for each possible value k). Consequently, Th_2_ can be computed (for a given value P_FS_ = **γ**) by using the following equations:

8 $${{\text{P}}}_{{\text{FS}}}={\int }_{{\text{k}}:{\text{L}}({\text{k}})<{{\text{Th}}}_{2}}{{\text{f}}}_{{\text{K}}}({\text{k}}; {{\text{H}}}_{0}){\text{dk}}=\upgamma ,$$

Consequently, the detection probability P_D_ can be calculated as

9 $${{\text{P}}}_{{\text{D}}}={\int }_{{\text{k}}:{\text{L}}({\text{k}})<{{\text{Th}}}_{2}}{{\text{f}}}_{{\text{K}}}({\text{k}}; {{\text{H}}}_{1}){\text{dk}}.$$

To estimate ${f}_{K}\left(k;{H}_{0}\right) \,and \,{f}_{K}\left(k;{H}_{1}\right)$, let ${f}_{I}\left(i;{H}_{0}\right) \,and \,{f}_{I}\left(i;{H}_{1}\right)$ represent the probability density function of a sample in ${\text{I}}\left[{\text{n}}\right],\mathrm{ n}\in [0,\mathrm{ N}-1]$ under H_0_ and H_1_ conditions, respectively. Given a threshold Th_1_ for the pre-threshold system (Eq. 5), we confirm that I_sw_ = i_sw_ [n] is a binary random variable with the values 0 or 1. Therefore, I_sw_, regarded as a result of the noise, leads to Escape (0), and the occurrence of IR@1550nm radiation power leads to Escape (1). This experimental setup is often referred to as the Bernoulli trial^87^. The probability ${\text{P}}\left({{\text{I}}}_{{\text{sw}}}=0; {{\text{H}}}_{0}\right)$ refers to the probability that I_sw_ = 0 given by H_0_. The probabilities ${\text{P}}\left({{\text{I}}}_{{\text{sw}}}=1; {{\text{H}}}_{0}\right),\mathrm{ P}\left({{\text{I}}}_{{\text{sw}}}=0; {{\text{H}}}_{1}\right)\mathrm{ and P}\left({{\text{I}}}_{{\text{sw}}}=1; {{\text{H}}}_{1}\right)$ are defined similarly and calculated as follows:

10 $${\text{P}}\left({{\text{I}}}_{{\text{sw}}}=0; {{\text{H}}}_{0}\right)={\int }_{{{\text{Th}}}_{1}}^{\infty }{{\text{f}}}_{{\text{I}}}\left({\text{i}}; {{\text{H}}}_{0}\right)\mathrm{ di }={{\text{a}}}_{0},$$

11 $${\text{P}}\left({{\text{I}}}_{{\text{sw}}}=1; {{\text{H}}}_{0}\right)={\int }_{-\infty }^{{{\text{Th}}}_{1}}{{\text{f}}}_{{\text{I}}}\left({\text{i}}; {{\text{H}}}_{0}\right)\mathrm{ di }={{\text{b}}}_{0},$$

12 $${\text{P}}\left({{\text{I}}}_{{\text{sw}}}=0; {{\text{H}}}_{1}\right)={\int }_{{{\text{Th}}}_{1}}^{\infty }{{\text{f}}}_{{\text{I}}}\left({\text{i}}; {{\text{H}}}_{1}\right)\mathrm{ di }={{\text{a}}}_{1},$$

13 $${\text{P}}\left({{\text{I}}}_{{\text{sw}}}=1; {{\text{H}}}_{1}\right)={\int }_{-\infty }^{{{\text{Th}}}_{1}}{{\text{f}}}_{{\text{I}}}\left({\text{i}}; {{\text{H}}}_{0}\right)\mathrm{ di }={{\text{b}}}_{1},$$

where ${{\text{a}}}_{0}+{{\text{b}}}_{0}=1,\mathrm{ and }{{\text{a}}}_{1}+{{\text{b}}}_{1}=1$. b_0_ and b_1_ are the probabilities of I_sw_ = 1 under H_0_ and H_1_ conditions, respectively. Considering that the IR@1550nm radiation is present, the detectable IR@1550nm signal amplitude is A_IR_ > 0, which can be verified. ${f}_{I}\left(i; {H}_{1}\right)$ is a left-side shift of ${f}_{I}\left(i; {H}_{0}\right),$ and the pre-threshold system is employed, and thus b_1_ > b_0_ is accurate. For ${\text{I}}\left[{\text{n}}\right],\mathrm{ n}=[0, 1,\dots ,{\text{N}}-1]$ under H_0_, we let $m\in [0, N]$ be the number of detecting IR@1550nm radiation signals (i.e. I_sw_ [n] = 1 when I [n] is applied to the pre-threshold system). The binomial distribution represents the discrete probability distribution of the number of occurrences of detecting IR@1550nm radiation power. The probability mass function associated with the variable m is denoted as ${f}_{M}\left(m\right)=\left(\genfrac{}{}{0pt}{}{N}{m}\right) {b}_{0}^{m} {a}_{0}^{N-m}$. Subsequently, we obtain:

14 $${{\text{f}}}_{{\text{K}}}\left({\text{k}}= \frac{1}{{\text{N}}}\sum_{{\text{n}}=0}^{{\text{N}}-1}{{\text{I}}}_{{\text{sw}}}\left[{\text{n}}\right]=\frac{{\text{m}}}{{\text{N}}}; {{\text{H}}}_{0}\right)= \left(\genfrac{}{}{0pt}{}{{\text{N}}}{{{\text{N}}}_{{\text{k}}}}\right) {{\text{b}}}_{0}^{{{\text{N}}}_{{\text{k}}}} {{\text{a}}}_{0}^{{\text{N}}-{{\text{N}}}_{{\text{k}}}},$$

where ${f}_{K}\left(k;{H}_{i}\right), i=\mathrm{0,1}$ is discrete because N and N_k_ are positive integers; k = m/N; m = 0, 1, …, N, the JRTC operating feature that has the shape of a staircase with the width of each rung varying with the value of N. If the value of N is high, then the staircase may be compacted, and the proof is exact. To avoid the discreteness impact of N, we utilise the continuous version of Eq. (14) for theoretical exposition, as follows:

15 $${{\text{f}}}_{{\text{K}}}\left({\text{k}}; {{\text{H}}}_{0}\right)=\frac{\mathrm{N \delta }({\text{N}}+1)}{\updelta \left({{\text{N}}}_{{\text{k}}}+1\right)\updelta ({\text{N}}-{{\text{N}}}_{{\text{k}}}+1)}{{\text{b}}}_{0}^{{{\text{N}}}_{{\text{k}}}} {{\text{a}}}_{0}^{{{\text{N}}-{\text{N}}}_{{\text{k}}}},$$

where ${\text{N}},{{\text{N}}}_{{\text{k}}}\mathrm{ \epsilon R}, 0\le {{\text{N}}}_{{\text{k}}}\le \mathrm{N and \delta }(.)$ is the Delta function defined as $\updelta \left({\text{N}}\right)={\int }_{0}^{\infty }{{\text{I}}}^{{\text{n}}-1} {{\text{e}}}^{-{\text{I}}}\mathrm{ dI}$. In Eq. (15), N is a scaling factor that causes the integral to be equal to 1. In a similar vein, we obtain:

16 $${{\text{f}}}_{{\text{K}}}\left({\text{k}}; {{\text{H}}}_{1}\right)=\frac{\mathrm{N \delta }({\text{N}}+1)}{\updelta \left({{\text{N}}}_{{\text{k}}}+1\right)\updelta ({\text{N}}-{{\text{N}}}_{{\text{k}}}+1)}{{\text{b}}}_{1}^{{{\text{N}}}_{{\text{k}}}} {{\text{a}}}_{1}^{{{\text{N}}-{\text{N}}}_{{\text{k}}}},$$

Using Eqs. (15) and (16), we can calculate the likelihood ratio test L (k) as follows:

17 $${\text{L}}\left({\text{k}}=\frac{{\text{m}}}{{\text{N}}}\right)={(\frac{{{\text{b}}}_{1}}{{{\text{b}}}_{0}})}^{{{\text{N}}}_{{\text{k}}}}{ (\frac{{{\text{a}}}_{1}}{{{\text{a}}}_{0}})}^{{{\text{N}}-{\text{N}}}_{{\text{k}}}},$$

Given that b_1_**/**b_0_ > 1 due to A_IR_ > 0, we have a_1_**/**a_0_ < 1. Thus, ${(\frac{{b}_{1}}{{b}_{0}})}^{{N}_{k}} \,and \,{ (\frac{{a}_{1}}{{a}_{0}})}^{{N-N}_{k}}$ rise monotonically as a function of k. Therefore, L (k) is consistently growing with respect to k, and the decision H_1_ is taken when k < Th_2_, which is determined by Eqs. (8) and (9)^47^. To determine Th_2_ threshold, the detection signals that correspond to each of the two hypotheses are set as follows: H_1_: K = m—n, and H_0_: K = n, where K is the test statistic value of the average switching current I_sw_[n] that is examined by the binary detection threshold Th_2_ (Fig. 5), and n is the Gaussian noise with zero mean and variance σ^2^. For hypothesis H_0_:

18 $${{\text{P}}}_{{\text{K}}|{{\text{H}}}_{0}}({\text{k}}|{{\text{H}}}_{0})=\frac{1}{\sqrt{2\pi }\sigma }\mathit{exp}\left(-\frac{{k}^{2}}{2{\sigma }^{2}}\right).$$

Consequently, the test statistic value K of the average I_sw_[n] under Hypothesis H_1_ is:

19 $${{\text{P}}}_{{\text{K}}|{{\text{H}}}_{1}}\left({\text{k}}|{{\text{H}}}_{1}\right)=\frac{1}{\sqrt{2\pi }\sigma }\mathit{exp}\left(-\frac{1}{2}\frac{{\left(k-m\right)}^{2}}{{\sigma }^{2}}\right).$$

The likelihood ratio is expressed as:

20 $$\Lambda \left({\text{t}}\right)=\frac{{{\text{P}}}_{{\text{K}}|{{\text{H}}}_{1}}({\text{k}}|{{\text{H}}}_{1})}{{{\text{P}}}_{{\text{K}}|{{\text{H}}}_{0}}({\text{k}}|{{\text{H}}}_{0})}=\mathit{exp}(-\frac{{m}^{2}-2km}{{2\sigma }^{2}}).$$

The likelihood ratio can be simplified by taking a logarithm of both sides of Eq. (20), which yields:

21 $${\text{ln}}\Lambda \left({\text{t}}\right)=\frac{m}{{\sigma }^{2}}k-\frac{{m}^{2}}{{2\sigma }^{2}} \begin{array}{c}{H}_{0}\\ \begin{array}{c}>\\ <\end{array}\\ {H}_{1}\end{array} \mathit{ln}\eta ,$$

where η = P(H_0_) **/** P(H_1_) is the minimum probability threshold. The comparable test after arranging terms is presented as:

22 $$\mathrm{K }\begin{array}{c}{{\text{H}}}_{0}\\ \begin{array}{c}>\\ <\end{array}\\ {{\text{H}}}_{1}\end{array} \frac{{\sigma }^{2}}{m}\mathit{ln}\eta + \frac{m}{2}= {{\text{Th}}}_{2}.$$

The decision regions (H_0_ and H_1_) are shown in Fig. 4, the probabilities of no signal (i.e. without IR radiation) and with the IR@1550nm radiation power are:

23 $${{\text{P}}}_{\mathrm{No \,signal}}=\mathrm{ P}\left(\mathrm{decide }{{\text{H}}}_{1}|{{\text{H}}}_{0}\mathrm{ \,true}\right)={\int }_{{Th}_{2}}^{\infty }\frac{1}{\sqrt{2\pi }\sigma }{{\text{e}}}^{\left(-\frac{{{I}_{sw}}^{2}}{2{\sigma }^{2}}\right)}d{I}_{sw},$$

and

24 $${{\text{P}}}_{\mathrm{with \,IR \,rad}.}=\mathrm{ P}\left(\mathrm{decide \,}{{\text{H}}}_{1}|{{\text{H}}}_{1}\mathrm{ \,true}\right)={\int }_{{Th}_{2}}^{\infty }\frac{1}{\sqrt{2\pi }\sigma }{{\text{e}}}^{\frac{-{\left({I}_{sw}-m\right)}^{2}}{2{\sigma }^{2}}}d{I}_{sw}.$$

respectively. Finally, the optimal decision rule under Hypothesis H_0_ (i.e. the noise alone induces escapes) is determined by:

25 $${{\text{P}}}_{{{{\text{I}}}_{{\text{sw}}}}_{{\text{K}}}|{{\text{H}}}_{0}}\left({{{\text{i}}}_{{\text{sw}}}}_{{\text{k}}}|{{\text{H}}}_{0}\right)=\frac{1}{\sqrt{2\pi }\sigma }\mathit{exp}\left(-\frac{{{{i}_{sw}}_{k}}^{2}}{2{\sigma }^{2}}\right),$$

where ${{i}_{sw}}_{k}$ is a sequence of test statistic values of the average switching current I_sw_[n]. Given that the Kth detection signal is assumed to be Gaussian with mean m and variance σ^2^, the optimum decision rule can be obtained as follows:

26 $${{\text{P}}}_{{{{\text{I}}}_{{\text{sw}}}}_{{\text{K}}}|{{\text{H}}}_{1}}\left({{{\text{i}}}_{{\text{sw}}}}_{{\text{k}}}|{{\text{H}}}_{1}\right)=\frac{1}{\sqrt{2\pi }\sigma }\mathit{exp}\left(-\frac{{{({i}_{sw}}_{k}-m)}^{2}}{2{\sigma }^{2}}\right).$$

Given that the noise samples (without IR radiation) are statistically uncorrelated, the joint density function of the K values is the product of each of the density functions:

27a $${{\text{P}}}_{{{\text{I}}}_{{\text{sw}}}|{{\text{H}}}_{0}}\left({{\text{i}}}_{{\text{sw}}}|{{\text{H}}}_{0}\right)=\prod_{{\text{k}}=1}^{{\text{K}}}\frac{1}{\sqrt{2\pi }\sigma }{e}^{\left(-\frac{{{{i}_{sw}}_{k}}^{2}}{2{\sigma }^{2}}\right)} ,$$

27b $${{\text{P}}}_{{{\text{I}}}_{{\text{sw}}}|{{\text{H}}}_{1}}\left({{\text{i}}}_{{\text{sw}}}|{{\text{H}}}_{1}\right)=\prod_{{\text{k}}=1}^{{\text{K}}}\frac{1}{\sqrt{2\pi }\sigma }{e}^{-\frac{{{({i}_{sw}}_{k}-m)}^{2}}{2{\sigma }^{2}}} ,$$

with Π representing the product, and ${\prod }_{{\text{k}}}{{\text{e}}}^{{\text{x}}}{\text{k}}= {{\text{e}}}^{{\sum }_{{\text{k}}}{{\text{x}}}_{{\text{k}}}}$. Consequently, the likelihood ratio becomes:

28 $$\Lambda \left({\text{t}}\right)=exp\left[\sum_{k=1}^{K}\frac{{{i}_{sw}}_{k}^{2}}{2{\sigma }^{2}}-\sum_{k=1}^{K}\frac{{({{i}_{sw}}_{k}-m)}^{2}}{2{\sigma }^{2}}\right]=\mathit{exp}\left[\frac{m}{{\sigma }^{2}}\sum_{k=1}^{K}{{i}_{sw}}_{k}-\frac{K{m}^{2}}{2{\sigma }^{2}}\right].$$

By applying the natural logarithm to both sides of the equation, the likelihood ratio becomes

29 $${\text{Ln}}\Lambda \left({\text{t}}\right)= \frac{m}{{\sigma }^{2}}\sum_{k=1}^{K}{{i}_{sw}}_{k}-\frac{K{m}^{2}}{2{\sigma }^{2}} \begin{array}{c}{{\text{H}}}_{0}\\ \begin{array}{c}>\\ <\end{array}\\ {{\text{H}}}_{1}\end{array} \mathit{ln}\eta .$$

After arranging the terms, we yield:

30 $$\sum_{{\text{k}}=1}^{{\text{K}}}{{{\text{i}}}_{{\text{sw}}}}_{{\text{k}}}\begin{array}{c}{{\text{H}}}_{0}\\ \begin{array}{c}>\\ <\end{array}\\ {{\text{H}}}_{1}\end{array} \left(\frac{{\sigma }^{2}}{m}\mathit{ln}\eta + \frac{Km}{2}\right)={{\text{Th}}}_{2},$$

where the JRTC is a device that aggregates K data samples and evaluates if their cumulative value is above the threshold Th_2_ or not, as determined by ${{\text{Th}}}_{2}=(\frac{{\sigma }^{2}}{m}\mathit{ln}\eta + \frac{Km}{2})$. When the prior probabilities are identical, the threshold is equivalent to one. Additionally, when using the log-likelihood ratio, the threshold is set at zero. Hence, this particular form effectively demonstrates the computation of posteriori probabilities, P(H_0_**|**I_sw_) and P(H_1_**|**I_sw_) to ascertain the detection of IR radiation power^85^. Subsequently, the hypothesis associated with the highest signal is selected^37,84^. The JRTC is designed by adhering to an optimal decision rule^88^, and the circuit calculates the probabilities of maximum switching current P(H_i_ **|** I_sw_), i = 0, 1, 2,…, M − 1. Subsequently, the JRTD detector selects the hypothesis associated with the highest switching probability as the preferred decision through the threshold control function. In Fig. 5, the threshold control functions are used for estimation and adjustment. Specifically, the pre-threshold system (Th_1_) is utilised to delineate and differentiate between the switching of the noise and the switching of arrival IR@1550nm radiation. Furthermore, the test statistic threshold (Th_2_) serves as the discriminant between hypotheses H_0_ and H_1_. In this study, we provide two distinct experimental distributions of switching probabilities that pertain to a specific collection of system characteristics. These distributions are differentiated based on the presence or absence of the IR@1550nm radiation power. According to Eq. (7), the superior value is determined by selecting the lower of the two values of α and β. In cases where the two distributions exhibit clear differentiation, identifying an appropriate threshold (Th_1_) becomes feasible. This threshold serves the purpose of achieving a low level of significance, such as α ≤ 1%, which effectively eliminates the likelihood that the observed outcomes are solely attributable to Gaussian noise. Additionally, Th_1_ is selected to ensure a high test power, specifically 1 − β ≥ 99%, thereby guaranteeing the detection of the IR@1550nm radiation signal^61^.

## Experimental setup

**Figure 6:**
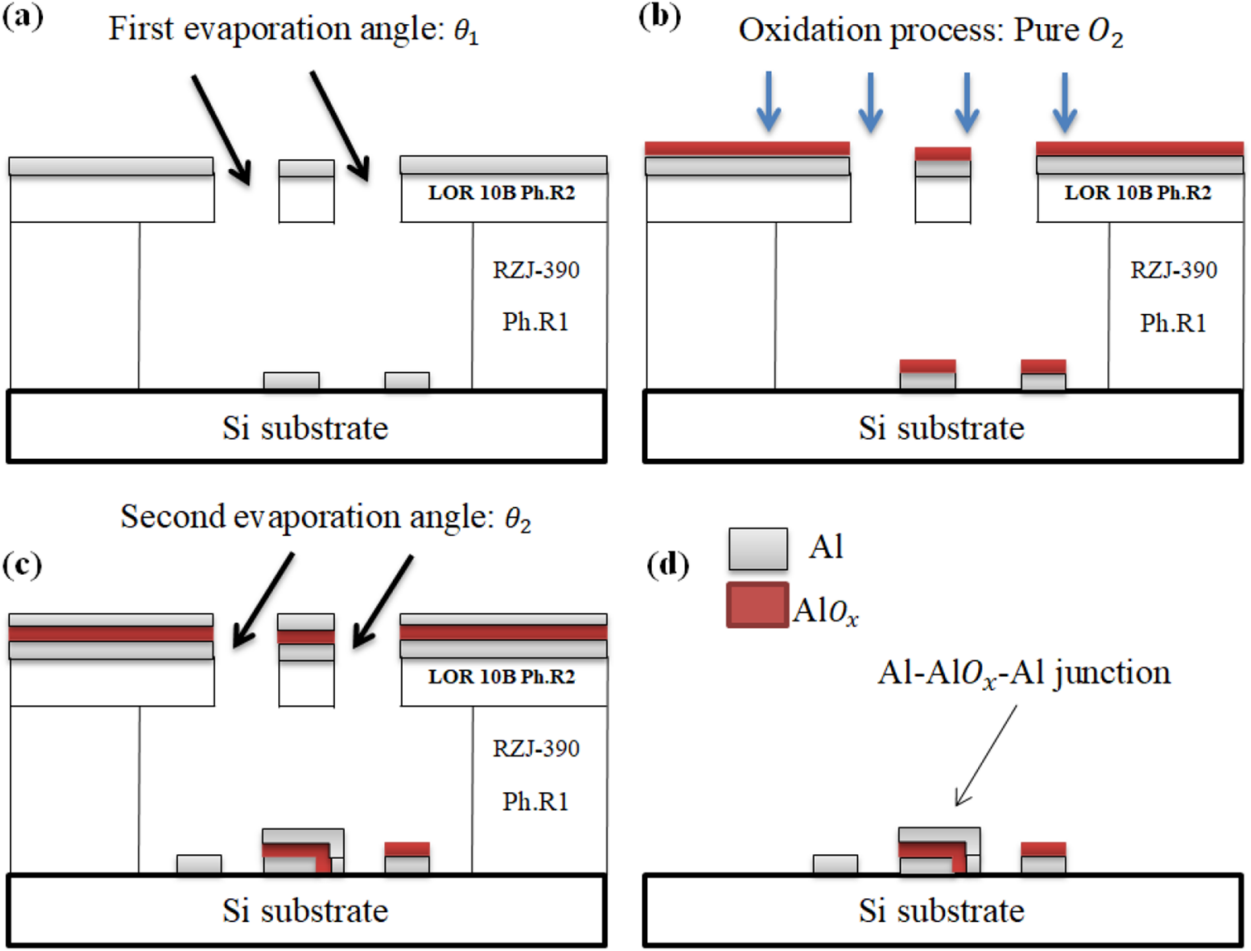
Schematic of the Al/AlO_x_/Al JJ fabrication process using electron beam double-angle evaporation techniques; (**a**) first evaporation angle (θ_1_ =  + 54°) to make the lower Al film, (**b**) Oxidation process to make the AlO_x_ layer, (**c**) second evaporation angle (θ_2_ =  − 54°) to make the upper Al film and (**d**) lift-off procedure.

The terminated Al/AlO_x_/Al junction is fabricated in our laboratory by using laser beam lithography (Microlab model) and electron beam double-angle evaporation techniques. Firstly, start with the Dolan bridge structure process: (Coating the bottom film—1st Photoresist (RZJ-390)—photolithography—development (RZX-3038)—etching (NaOH)—degumming—2nd Photoresist (LOR 10B)—photolithography—development (RZX-3038)). Secondly, the JJ fabrication process: Place the sample in the coating machine, in the position of the first evaporation angle (θ_1_ =  + 54°), then begin coating the lower Al film (thickness = 100 nm). The AlO_x_ layer was obtained by oxidising the top of the first Al film in pure oxygen at a pressure of 3.2 mbar for 10 min^89^. Then, switch the sample position to the second evaporation angle (θ_2_ =  − 54°) and start coating the upper Al film (thickness = 120 nm), as shown in Fig. 6. The fabricated JJ area is approximately 30 μm^2^, and the junction has been built on a 0.5 mm-thick, 6 mm × 6 mm silicon substrate. Figure 1 shows our experimental setup for the proposed JRTD measurements; The measured JJ is placed in an aluminium sample box (27.5 mm long, 26.5 mm wide, and 11 mm thick), and the box is placed in a dilution refrigerator (Leiden Cryogenics B.V.; CRYOGEN-FREE DILUTION REFRIGERATOR, CF-CS50 model) at the mixing chamber. To prevent and dampen thermal noise, twisted-pair copper cables are used to link the AC and RF lines. At a temperature of 42 mK, the IV curve of the device is measured by using four probe methods. The measurements revealed non-hysteretic IV curve behaviour (Fig. 3a), because of small JJ capacitance, thereby causing the McCumber–Stewart damping parameter to be less than unity (β_c_ < 1)^90^. The function generator can generate a sinusoidal voltage signal, which is applied to the bias resistor (100 KΩ) to drive the bias current, I_b_ through the junction. All electrical lines that link the sample box to the operating electronics at room temperature 300 k are filtered by using RC low-pass filters (home-made RC filters) with a cutoff frequency of 10 kHz. A laser diode source (S/N: 141,014-10) is used to provide a consistent infrared radiation beam with a wavelength of 1550 nm.

The JJ is lighted using a single-mode optical fibre cable (SMF-28), and the IR@1550nm radiation that passes through an optical radiation attenuator (8156A OPTICAL ATTENUATOR model) has regulated attenuation from 740 nW to the minimum obtainable radiation power 0.74 pW. The IR@1550nm radiation power is focused onto the upper surface of the JJ superconducting electrode by means of a hole located in the lid of the sample box that contains the JJ at the mixing chamber of the dilution refrigerator. Meanwhile, the output signals are passed via a LPF (RC) to separate the noise component. Subsequently, they are amplified by a power amplifier (home-made) with a gain of 10^3^ before being inputted into the JRTC for the binary hypothesis detection process (Fig. 5).

## Results and discussions

**Figure 7:**
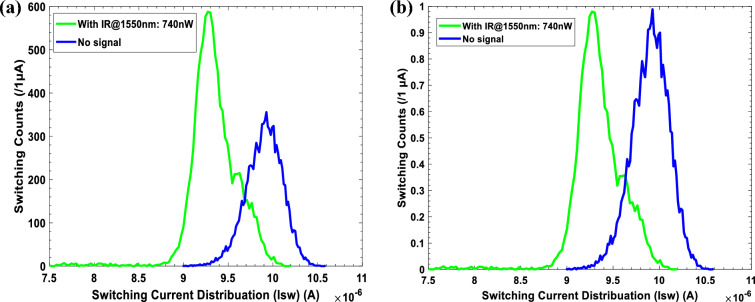
Measured switching current distributions: (**a**) blue refers to the case without radiation ‘No signal’, and green refers to the case with IR@1550nm radiation of the power being 740 nW. (**b**) normalised histograms of the switching current distribution in (**a**). In this work, I_b_ is changed gradually from 0 to 25 µA with the repetition frequency being 71.3 Hz, the resistance controlling JJ bias R = 100 KΩ and laser diode drive voltage = 1.2 V, and each histogram accumulates 10^4^ switching events (/1 µA).

The statistics of photon noises govern the seeming uncontrolled switching with IR@1550nm radiation signal. We created a histogram of switching occurrences in Fig. 7a, as predicted for uncorrelated switching events. A Poisson distribution of the switching current distribution, I_sw_ is shown, for two cases: (i) no signal, see the blue histogram, and the peak is at 9.95 μA (this case mimics the hypothesis H_0_, i.e., the noise alone induces escapes), and (ii) under IR@1550nm radiation with the power 740 nW, see the green histogram, and the peak is at 9.34 μA (this case mimics Hypothesis H_1_, the noise and radiation induce the escapes). The noisy environment (i.e., Gaussian noise and measurement noise) affects the histogram of switching current distributions without IR radiation ‘No signal’. Meanwhile, the width of the green curve is the same as that of the blue curve, but the edges of the latter are sharp and vibrated (Fig. 7b).

Statistical decision theory^41,69^ is used to examine the observed data for the switching current distribution to determine an appropriate decision rule for our proposed JRTD. To achieve the optimum design of the JRTD, one determines the probability P_0_ (I_sw_) and P_1_ (I_sw_) that a switch will occur after and before the threshold Th_1_ for each of the binary hypotheses H_0_, and H_1_, respectively. To determine the probability of switching events:

31a $${\text{P}}\left({\left({{\text{I}}}_{{\text{sw}}}\right)}_{0}\right)={\int }_{{{\text{Th}}}_{1}}^{\infty }{\text{P}}{\left({{\text{I}}}_{{\text{sw}}}\right)}_{0}\mathrm{ d}{{\text{I}}}_{{\text{sw}}} ,$$

31b $${\text{P}}\left({\left({{\text{I}}}_{{\text{sw}}}\right)}_{1}\right)={\int }_{-\infty }^{{{\text{Th}}}_{1}}{\text{P}}{\left({{\text{I}}}_{{\text{sw}}}\right)}_{1}\mathrm{ d}{{\text{I}}}_{{\text{sw}}} ,$$

where the switching probability with ‘no signal’ is ${\text{P}}{\left({{\text{I}}}_{{\text{sw}}}\right)}_{0}$, and ${\text{P}}{\left({{\text{I}}}_{{\text{sw}}}\right)}_{1}$ is the switching probability in the presence of an IR radiation@1550nm with the power set to 740 nW. Solve Eq. (31) by substituting ${\text{P}}{\left({{\text{I}}}_{{\text{sw}}}\right)}_{\mathrm{0,1}}$ from Eqs. (3) and (4). We can write the two possible hypotheses (H_0_ | H_1_) probabilities as follows:

32a $${\text{P}}\left({{\text{H}}}_{0}\right)=\frac{1}{\sqrt{2\pi }{\sigma }_{0}}{\int }_{{{\text{Th}}}_{1}}^{\infty }{e}^{\frac{{-\left({I}_{sw0}-{\mu }_{0}\right)}^{2}}{2{\sigma }_{0}^{2}}} d{I}_{sw} ,$$

32b $${\text{P}}\left({{\text{H}}}_{1}\right)=\frac{1}{\sqrt{2\pi }{\sigma }_{1}}{\int }_{-\infty }^{{{\text{Th}}}_{1}}{e}^{\frac{{-\left({I}_{sw1}-{\mu }_{1}\right)}^{2}}{2{\sigma }_{1}^{2}}} d{I}_{sw}.$$

**Figure 8:**
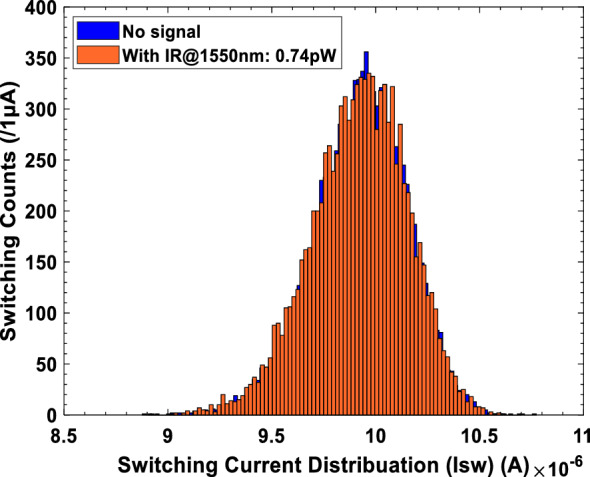
Measured switching current distributions: Blue refers to the case without radiation, and orange refers to the case with IR@1550nm radiation power 0.74 pW. Here, I_b_ is changed gradually from 0 to 25 µA with the repetition frequency being 71.3 Hz, the resistance controlling JJ bias R = 100 KΩ, laser diode drive voltage = 1.2 V, and each histogram accumulates 10^4^ switching events (/1µA).

The output of the JRTC exhibits two distinct scenarios: In the first scenario, we consider the null hypotheses: H_0_, Eq. (32a) indicating the switching occurred in the absence of the radiation signal, given a measured value of I_sw0_ = 9.95 µA, surpassing thresholds Th_1_ and Th_2_. In the subsequent scenario, we consider alternative hypothesis H_1_, and Eq. (32b) suggests that the switching occurred in the presence of IR radiation@1550nm with the power 740 nW, and the measured value I_sw1_ = 9.34 µA, is smaller than those of thresholds Th_1_ and Th_2_. The suggested JRTD aims to determine the optimal decision by formulating certain hypotheses that are derived from the switching probabilities of the received IR radiation signals. The threshold switching current Th_1_ for the transition between states H_0_ and H_1_ was measured as 9.72 μA. This measurement was conducted in the absence of radiation, and under the influence of a 740 nW IR@1550nm radiation power. The limit of the switching current induced by Gaussian noise: H_0_ is ${{({\text{I}}}_{{\text{sw}}})}_{{\text{Null}}}>9.72 \mu {\text{A}}$. Similarly, the switching current induced by the Gaussian noise and the arrival of IR@1550nm radiation power H_1_ is ${{({\text{I}}}_{{\text{sw}}})}_{{\text{Alter}}.}<9.72 \mu {\text{A}}$. Furthermore, the detection of the switching current in response to IR radiation is within the range of ${{8.59 \mu {\text{A}}<({\text{I}}}_{{\text{sw}}})}_{{\text{Alter}}.}<9.72 \mu {\text{A}}$. Based on the findings obtained from these experimental results, the minimum obtainable radiation power of our instrumentation is 0.74 pW. Additionally, the corresponding minimum switching current is ${{({\text{I}}}_{{\text{sw}}})}_{{\text{Min}}}=9.91 \mu {\text{A}}$, as seen in Fig. 8. Meanwhile, the noise results in the generation of escape amounts α = 8.99 µA, whereas the noise combined with the arrival of IR@1550nm radiation power leads to *β* = 10.40 µA. These values of α and β were extracted directly from the figure properties window in the 'MATLAB' software that we used to generate the switching current histograms.

**Figure 9:**
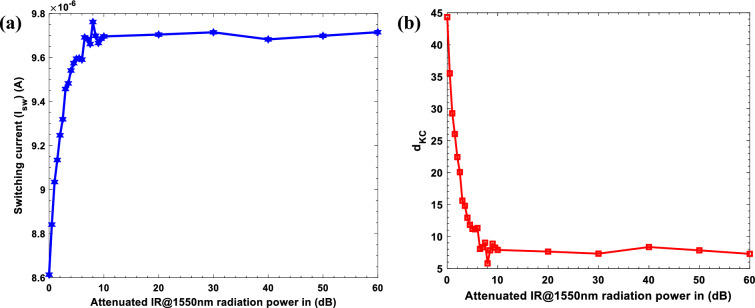
(**a**) Experimental data of switching current distribution vs. attenuated IR@1550nm radiation power in dB, show a linear relationship between the switching current and the attenuated IR radiation beam power in the window of 740 nW (0 dB) to 74 nW (10 dB), and stability in the curve for the values above 74 nW (10 dB) to the minimum obtainable radiation power of our instrumentation 0.74 pW (60 dB). (**b**) KC-index from Eq. (33) as a function of attenuated IR@1550nm radiation power in dB shows a maximum value (44.3) of d_KC_ at 740 nW (0 dB), which confirms the results in (**a**).

The optimal design of JRTD is supposed to detect a single IR photon energy, E = h/cλ; where E is the photon energy, h is Planck’s constant, λ is the wavelength, and c is the speed of light in vacuum. This energy is calculated as 0.7999 eV (1.28 × 10^−19^ J)^91,92^. The minimum detectable power of the proposed JRTD can be estimated as 0.74 pW by reducing the IR radiation power to the minimum obtainable radiation power of our instrumentation that causes JJ transition. We achieved this process by using an optical radiation attenuator, coupled in series with the IR radiation source. This attenuator reduces the IR radiation power from 740 nW to 0.74 pW by adding a calibrated amount of power loss in decibel units (0–60 dB) to the IR radiation generated by the IR radiation source. Figure 9a illustrates the relationship between the attenuated IR@1550nm radiation and the switching current distributions by gradually decreasing the IR radiation power from 740 nW to 0.74 pW, which is comparable with the attenuator range from 0 to 60 dB whilst maintaining the same junction under test but with different measurement parameters. The linear relationship between the switching current and the attenuated IR radiation power within the range of 0 dB to 10 dB, equal to 740 nW to 74 nW, is evident. The switching current varies from 8.6 µA to 9.696 µA, including an average range of 1.096 µA. After this region (10 dB), the curve stability for the IR radiation power lower than 74 nW (10 dB) to the minimum radiation level applied from the IR radiation source, which is 0.74 pW (60 dB), the equivalent switching current values varied from 9.696 µA to 9.715 µA. Despite the small displacement that occurred in the values of the switching currents, the minimum obtainable radiation power of our instrumentation (0.74pW) is also obtained in this different measurement method.

The JRTD minimum detectable power depends on the junction characteristics, the IR radiation source and the attenuator loss gain. Our studies were performed with a minimum detectable power of 0.74 pW, and the rate of detecting photons from IR radiation was 5.692 × 10^6^ photons per second according to; R_ph_ = P_min_ / E, where R_ph_ is the photon rate, P_min_ is the minimum detectable power, and E = hv is the energy of each photon. Based on these results, the presence of noise in the transmission of the IR@1550nm radiation has an effect on the precision of measurements and the value of the switching current. For computing the JRTD performance parameters, we evaluate the minimum attainable sensitivity of the JRTD as the IR radiation signal required to equal the minimum JRTD noise power (Noise equivalent power (NEP)) according to; ${\text{NEP}}/\Delta V=\sqrt{6}{\left(\frac{\mathrm{\hbar }{\omega }_{{\text{IR}}}}{2{{\text{eR}}}_{{\text{J}}}{{\text{I}}}_{{\text{c}}}}\right)}^{2}\sqrt{4{k}_{B}T{R}_{J}}$ , where ω_IR_ is the IR radiation frequency, k_B_ is Boltzmann constant, and T is the working temperature^93,94^. The NEP estimated to be (**~ **2.85 × 10^−13^ W Hz^−1/2^) is a clear indication of the high sensitivity of the proposed JRTD. Moreover, to estimate the performance of the JRTD, we calculated the Kumar-Caroll (KC) index d_KC_, which is a signal-to-noise ratio (SNR) indicator^95^. We obtained that through the analysis of the switching currents (I_sw_) of the JJ transition with and without IR@1550nm illumination as follows:

33 $${{\text{d}}}_{KC}=\frac{\left|{\langle {I}_{sw}\rangle }_{1}- {\langle {I}_{sw}\rangle }_{0}\right|}{\sqrt{\frac{1}{2}[{\sigma }^{2}{\left({I}_{sw}\right)}_{1}+{\sigma }^{2}{\left({I}_{sw}\right)}_{0}]}} ,$$

where ${\langle {I}_{sw}\rangle }_{1}=\frac{1}{N}\sum_{i=1}^{N}{{I}_{sw}}_{i}{|}_{1}$ is the average value of the I_sw_ in the presence of the IR@1550nm radiation power and noise, ${\langle {I}_{sw}\rangle }_{0}$ describes the I_sw_ in the absence of the IR@1550nm radiation and the presence of the noise alone, and ${\sigma }^{2}{\left({I}_{sw}\right)}_{1}=\frac{1}{N(N-1)} \sum_{i=1}^{N}{({{I}_{sw}}_{i}-{\langle {I}_{sw}\rangle }_{1})}^{2}$ is the variance of the average I_sw_^96^. Figure 9b shows the d_KC_ index analysis, which agrees very well with the experimental results of switching current distribution vs. attenuated IR@1550nm radiation power in dB. We can observe that the d_KC_ parameter values are higher in the window of 740 nW (0 dB) to 74 nW (10 dB), which confirms the experimental results in Fig. 9a. We conclude that the proposed JRTD can detect lower IR radiation if radiation source lower than 0.74 pW is provided, and the Gaussian noise is further suppressed.

## Conclusions

In summary, we designed and fabricated the JRTD that utilises the CBJJ at cryogenic temperatures, thereby serving as an infrared radiation sensor capable of binary detection. The binary hypothesis detection problem can be effectively addressed by utilising the JRTC. Furthermore, we have conducted an experimental analysis on the switching current distributions of the Al/AlO_x_/Al CBJJ to investigate the anticipated responses of the JRTD. Considering the effects of the Gaussian noise, a method based on standard statistical decision theory^84,96^ is developed to process the accumulated data of the switching current distributions for calibrating threshold control functions (Th_1_ and Th_2_). The results prove that the device can be used for the binary detection of the photons. Given the quality of the fabricated CBJJ can be further improved, and the numerical approaches to processing the detected data can also be further optimised, and the proposed JRTD based on the CBJJs can be used to implement the sensitive detection of photons (NEP ~ 2.85** × **10^−13^ W Hz^−1/2^) in a noisy environment. Furthermore, our results suggest a potential to enhance the minimum detectable power of the suggested JRTD detector by using radiation source lower than 0.74 pW and reducing the Gaussian noise of the junction. Finally, the JRTD performance is evaluated through the SNR indicator (d_KC_), which shows a maximum value of 44.3 at 740 nW, which confirms our experimental results.

## Data Availability

The datasets used and/or analysed during the current study are available from the corresponding author upon reasonable request.
