# Author Correction: Josephson radiation threshold detector

**DOI:** 10.1038/s41598-024-56375-w

**Published:** 2024-03-11

**Authors:** Soragga Ali, P. H. Ouyang, J. X. He, Y. Q. Chai, L. F. Wei

**Affiliations:** Information Quantum Technology Laboratory, International Cooperation Research Center of China Communication and Sensor Networks for Modern Transportation, School of Information Science and Technology, Southwest Jiaotong University, Chengdu, 610031 China

Correction to: *Scientific Reports* 10.1038/s41598-024-52684-2, published online 30 January 2024

In the original version of this Article, P. H. Ouyang was incorrectly listed as a corresponding author. The correct corresponding author for this Article is L. F. Wei. Correspondence and request for materials should be addressed to lfwei@swjtu.edu.cn.

The original Article has been corrected.

